# Comparative Proteomic Analysis Provides New Insights into the Development of Haustorium in Taxillus chinensis (DC.) Danser

**DOI:** 10.1155/2022/9567647

**Published:** 2022-07-28

**Authors:** Limei Pan, Lingyun Wan, Lisha Song, Lili He, Ni Jiang, Hairong Long, Juan Huo, Xiaowen Ji, Fengyun Hu, Jine Fu, Shugen Wei

**Affiliations:** ^1^Guangxi Botanical Garden of Medicinal Plants, Nanning 530023, China; ^2^Guangxi Key Laboratory for High-Quality Formation and Utilization of Dao-di Herbs, China

## Abstract

Taxillus chinensis is an important medicinal and parasitic plant that attacks other plants for living. The development of haustorium is a critical process, imperative for successful parasitic invasion. To reveal the mechanisms underlying haustorium development, we performed an iTRAQ-based proteomics analysis which led to the identification of several differentially abundant proteins (DAPs) in fresh seeds (CK), baby (FB), and adult haustoria (FD). A total of 563 and 785 DAPs were identified and quantified in the early and later developmental stages, respectively. Pathway enrichment analysis revealed that the DAPs are mainly associated with metabolic pathways, ribosome, phenylpropanoid biosynthesis, and photosynthesis. In addition, DAPs associated with the phytohormone signaling pathway changed markedly. Furthermore, we evaluated the content of various phytohormones during different stages of haustoria development. These results indicated that phytohormones are very important for haustorium development. qRT-PCR results validated that the mRNA expression levels were consistent with the expression of proteins, suggesting that our results are reliable. This is the first report on haustoria proteomes in the parasitic plant, Taxillus chinensis, to the best of our knowledge. Our findings will enhance our understanding of the molecular mechanism of haustoria development.

## 1. Introduction

Taxillus chinensis (DC.) Danser is a member of the family Loranthaceae which consists of approximately 73 genera and 900 species and is majorly comprised of aerial hemiparasitic plants [1]. T. chinense is geographically distributed in the southern and southwestern parts of China. It is an important plant in traditional Chinese medicine (TCM), mainly because its stem and leaves can be used for the treatment of rheumatoid arthralgia, risk of spontaneous abortion, and hypertension [2]. The host plants of T. chinensis include mulberry, peach, plum, longan, litchi, carambola, camellia, and other economically important plant species [3]. T. chinensis absorbs mineral nutrients and water from the host plant through a unique multicellular organ termed the haustorium that mediates the establishment of host-parasite interaction. Though they provide carbohydrates through photosynthesis which is essential for its growth [1, 4].

The initiation of haustorium development in most parasitic plants is triggered by host-derived chemical signals released after seed germination. These components include phenolic acids, flavonoids, quinones, and 2,6-dimethoxy-p-benzoquinone (DMBQ) [5]. However, the precise mechanism of how these compounds are released and trigger haustorium development is not well understood. Few of the genes have been identified to date that may be involved in these processes. For instance, TvQR1 encoding a quinone oxidoreductase trigger haustoria development in the facultative parasite Triphysaria versicolor [6, 7]. The possible reason could be that this enzyme converts quinones to semiquinones which serve as intermediate products in redox cycling. Semiquinone itself or redox cycling might be involved in the haustorium induction pathway [6, 8]. A recent model proposes that phenolic acids derived from the host cell wall degradation are oxidized by ROS and oxidative enzymes, generating haustorium-inducing factor (HIF) quinones [9, 10]. Besides, it has also been proved that the accumulation of auxin and ethylene acts as early events during haustorium development in the hemiparasitic plant T. versicolor [11]. The significant depositions of cytokinins (CKs), zeatin (Z), zeatin riboside (ZR), zeatin nucleotide (ZN), and ABA were detected in the haustoria of Rhinanthus–Hordeum vulgare association [12, 13]. The seeds of Santalum album, an aggressive root hemiparasite, can be germinated in the sand or in vitro on the Murashige and Skoog (MS) medium after pretreatment with 2-8 mM GA3 for 12 h and then develop the haustoria within one month without the need for induction with HIFs [14–16]. These results suggest that the plant hormones play a crucial role in the regulation of haustorium development.

Rapid advancement in the microarray, next-generation sequencing, and mass spectrometry- (MS-) based technologies has enabled high throughput analyses of transcriptomes, proteomes, and metabolomes, serving as powerful tools for obtaining large-scale information on transcripts, proteins, and metabolites. Earlier, the transcriptome of different stages during haustoria formation in T. chinense has been investigated which revealed the possible mechanism of haustorial development [2]. Study of biological processes at the protein level provides more realistic information because the proteins but not the mRNAs are functional and posttranslational processes result in the production of different protein isoforms [17]. Previous studies have focused on the mechanism of dehydration stress response in the T. chinense using proteomics analysis [18]. However, there are few reports available to date on the proteomic analysis of haustorial development in T. chinense [18]. The current study will provide novel insights into the molecular basis of haustorial development and help to fill these gaps in our knowledge.

## 2. Methods

### 2.1. Plant Material

The fresh seeds of Taxillus chinensis (DC.) Danser were collected from the parasitized mulberry trees that were planted in the experimental field of Guangxi Botanical Garden of Medicinal Plants, Guangxi, China. Seeds were accessed by the senior botanists and deposited in the herbarium of Guangxi Botanical Garden of Medical Plants (accession number: s0001794). The seeds were peeled, washed with sterile water, placed on a germination dish, and incubated in a controlled environment (25°C, 80% relative humidity, 10 h: 14 h light: dark, 2000 Lx), as previously described [2]. The fresh seeds were collected as a control (CK). The seeds with protruding seed-type radicles and tiny suction devices were collected after ten days of incubation (FB). Twenty days later, seeds with the Taxillus chinensis haustoria and true leaves were collected (FD). All the samples were immediately frozen in liquid nitrogen and stored at -80°C for subsequent experiments.

### 2.2. Protein Extraction

Total protein was extracted as described in a previous study [19]. Briefly, the seed samples were frozen in liquid nitrogen and pulverized. The samples were mixed with 5 volumes of chilled acetone containing 10% (*v*/*v*) trichloroacetic acid (TCA) and incubated at -20°C overnight. After centrifugation at 6,000 g for 40 min, the supernatant was discarded. The precipitant was washed thrice with precooled acetone and air-dried. Pellet was dissolved in the Lysis buffer (8 M urea, 120 mM NaCl, 10 mM EDTA, 1% Triton X100, and 1% PMSF dissolved in 50 Mm Tris-HCl pH 8.0). Finally, the suspension was filtered through 0.22 mm filters after centrifugation at 13,000 g for 10 min at 4°C. The concentration of protein was quantified with the BCA Protein Assay Kit (Bio-Rad, USA), and the quality of the protein sample was measured by SDS-PAGE.

### 2.3. Protein Digestion and iTRAQ Labeling

For each sample, 100 *μ*g of total protein was used for trypsin-based digestion followed by iTRAQ labeling. Protein was reduced with 10 mM DTT at 37°C for 60 min and alkylated with 55 mM iodoacetamide (IAM) at room temperature for 30 min in the dark. The urea concentration of the protein samples was diluted to less than 2 M by adding 100 mM TEAB. Next, the trypsin was added to the protein pool of each sample with the ratio of protein : trypsin = 50 : 1 (mass ratio) at 37°C overnight and 100 : 1 for a second digestion for 4 h. After trypsin digestion, peptides were desalted with the Strata X SPE column and vacuum-dried. The peptides were reconstituted in 20 *μ*L of 500 mM TEAB and labeled according to the manufacturer's protocol. Briefly, the peptide solution was mixed with 50 *μ*L isopropanol, and one unit of iTRAQ reagent was added to each sample. The mixture was incubated for 2 h at room temperature, pooled, and dried by vacuum centrifugation.

### 2.4. High-Performance Liquid Chromatography (HPLC) Fractionation

The peptides were reconstituted in the HPLC solution A (2% ACN, pH 10.0) and fractionated using high pH reverse-phase HPLC with Waters Bridge Peptide column BEHC18 (130 Å, 3.5 *μ*m, and 4.6∗250 mm). Briefly, peptides were first separated into 72 fractions using a gradient of 2% to 98% acetonitrile (pH 10). The wavelength 250 nm is used for the detection of peptides. Then, the peptides were pooled into 18 fractions and dried by vacuum centrifugation. The peptide fractions were desalted using Ziptip C18 (Millipore, Billerica, MA) according to the manufacturer's instructions.

### 2.5. Liquid Chromatography Coupled with the Tandem Mass Spectrometry (LC-MS/MS) Analysis

Mass spectrometry-based analysis was performed by NanoLC 1000 LC-MS/MS using a Proxeon EASY-nLC 1000 coupled with Thermo Fisher Q Exactive. Digested fractions were resuspended using 0.1% formic acid and loaded onto a reversed-phase-column (Acclaim PepMap®100 C18, 3 *μ*m, 100 Å, 75 *μ*m × 2 cm) at a rate of 5 *μ*L/min in 100% solvent A (0.1 M acetic acid in water). Next, peptides eluted from the trap column were loaded onto a reversed-phase analytical column (Acclaim PepMap®RSLC C18, 2 *μ*m, 100 Å, 50 *μ*m × 15 cm). The gradient was comprised of solvent B (0.1% FA in 98% ACN) with an increasing concentration from 15% to 35% over 45 min, 35% to 98% for 5 min, and kept in 98% solvent B for 5 min at a constant flow rate of 300 nl/min on an EASY-nLC1000 system. The elute was sprayed via NSI source at the 2.0 kv electrospray voltage and analyzed by tandem mass spectrometry (MS/MS) in Q Exactive. A data-dependent acquisition mode in the scan range of 350-2000 m/z was carried out for the mass spectrometry analysis, and the survey scans were captured by the Orbitrap analyzer at a mass resolution of 17500. In the linear ion trap, 15 of the most intense precursor ions were selected for subsequent decision tree-based ion trap HCD fragmentation at the normalized collision energy of 32% in the MS survey scan with 10.0 s dynamic exclusion.

### 2.6. Raw Data Processing

The raw data files were searched against the transcriptome database using Mascot software integrated into the Proteome Discoverer (Thermo Scientific, USA). The search parameters were used as follows: carbamidomethylation (C) was set as fixed modifications, whereas oxidation (M), and acetylation at the N-Term were set as variable modifications. Trypsin was chosen as the enzyme, and two missed cleavages were allowed. Mass tolerance of precursor ions was set as 20 ppm, and a fragment ion tolerance was 0.05 Da, resulting in a 1% false discovery rate (FDR). Differentially abundant proteins (DAPs) were identified based on the following criteria: *P* values less than 0.05 and a mean relative abundance > 1.2 or <0.83.

### 2.7. Bioinformatics Analysis

To determine the functional characterization of differentially abundant proteins (DAPs), proteins were mapped with Gene Ontology (GO) annotation base on UniProt-GOA database (http://www.ebi.ac.uk/GOA) [20]. All the proteins were grouped into three major categories: biological processes, cellular components, and molecular functions. The metabolic pathway analysis of DAPs was based on the Kyoto Encyclopedia of Genes and Genomes (KEGG) database (http://www.genome.jp/kegg/) [21].

### 2.8. Phytohormone Quantification

Phytohormone indole-3-acetic acid (IAA), gibberellin (GA), and abscisic acid (ABA) were quantified using liquid chromatography coupled with a mass spectrometry system (LC-MS, 1120-6460, Agilent, USA). Phytohormone extraction was performed from the Taxillus chinensis seeds based on the previous method [18]. The phytohormone was separated by a C18 column (Hypersil Gold, 100 mm × 2.1 mm, 1.9 *μ*m, Thermo Fisher Scientific) at a flow rate of 0.3 mL/min with a 17 min gradient elution. For multiple reaction monitoring (MRM), the phytohormones were analyzed with the negative mode by the ESI ion source.

### 2.9. Quantitative Real-Time PCR Analysis

Total RNA was extracted from each sample using TRIzol reagent (Invitrogen), following the manufacturer's protocol. FastQuant RT Kit (with gDNase, Tiangen) was used for DNA removal and cDNA synthesis. Specific primer pairs from randomly selected 8 genes were designed for quantitative real-time PCR (qRT-PCR) using the Primer Premier 5.0 software. Sequences of the primers used in this study are given in Table S1. Actin was used as the reference gene (3). The procedure of the qRT-PCR experiment was the same as our previous study [22]. The 2^−*ΔΔ*Ct^ method was used to evaluate the expression levels of transcripts in each sample [23]. Each experiment was performed with three biological replicates and three technical replicates.

### 2.10. Statistical Analyses

Statistical comparisons were made using the SPSS statistics software package with a significance level of *P* < 0.05.

## 3. Result

### 3.1. Differentially Abundant Protein (DAP) Analysis

**Figure 1:**
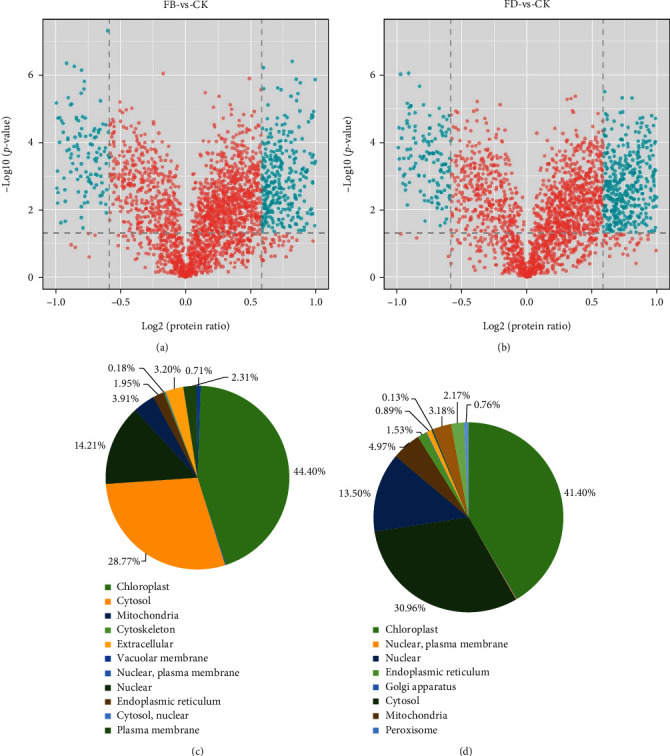
Identification of differentially abundant proteins (DAPs) between FB vs CK (a) and FD vs. CK (b). Subcellular locations of differentially abundant proteins (DAPs) between FB vs. CK (c) and FD vs. CK (d).

To investigate the potential mechanism involved in the haustorial development in T. chinense, an integrated approach involving LC-MS/MS and iTRAQ labeling was applied to analyze the proteomic changes. After processing MS/MS spectra in the Mascot software, a total of 325354 spectra were generated, which were segregated into 26463 matched spectra, 24367 unique spectra, 10325 matched peptides, 9462 unique peptides, and 2615 matched proteins (Figure S1). A total of 563 proteins were identified as DAPs between FB treatment and the control, of which 384 were identified as upregulated and 179 were downregulated proteins, respectively. In addition, a total of 785 proteins were identified as DAPs between FD treatment and control, of which 569 and 216 were identified as upregulated and downregulated proteins, respectively (Figure 1). All the DAPs were grouped based on their subcellular localization. For the FB treatment, proteins localized in 11 subcellular components were identified, including 250 chloroplast-localized proteins (44.4%), 162 cytosol-localized proteins (28.77%), and 80 nuclear-localized proteins (14.21%). For the FD treatment, proteins localized in 16 subcellular components were identified, including 325 chloroplast-localized proteins (41.4%), 2433 cytosol- localized proteins (30.96%), and 106 nuclear-localized proteins (13.5%) (Figure 1).

### 3.2. Functional Categorization Analysis

**Figure 2:**
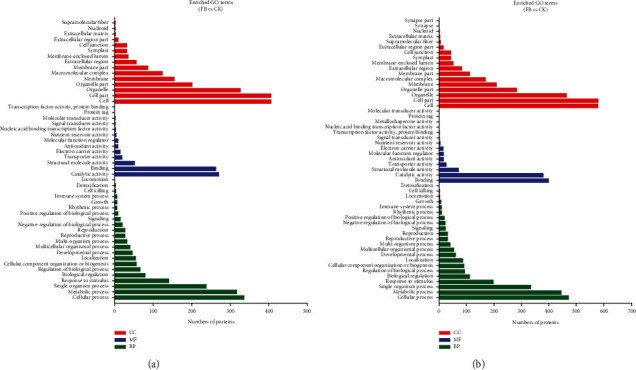
Gene Ontology (GO) analysis of differentially abundant proteins (DAPs) between FB vs. CK (a) and FD vs. CK (b). And the proteins were annotated by biological process, cellular component, and molecular function.

The DAPs were classified into three main GO categories (cellular component, biological process, and molecular function). In the early developmental stage (FB), 1546 DAPs (some proteins have more than one GO annotation) were annotated with biological process, 653 DAPs with molecular functions, and 1885 DAPs with cellular components compared to control. In the later developmental stage (FD), 2126 DAPs were annotated with biological process, 945 DAPs with molecular functions, and 2665 DAPs with cellular components compared to control. In the biological process, the most enriched categories were the cellular process and metabolic process. In the molecular function, the most abundant category was binding and catalytic activity. In the cellular component, term cell and cell part were found to be highly enriched (Figure 2).

### 3.3. Metabolic Pathway Analysis

**Figure 3:**
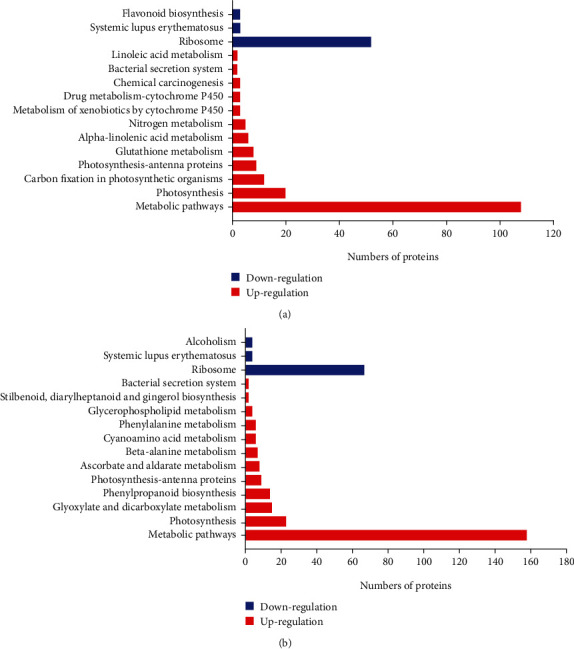
Kyoto Encyclopedia of Genes and Genomes (KEGG) analysis of differentially abundant proteins (DAPs) between FB vs. CK (a) and FD vs. CK (b).

To further understand the molecular mechanism potentially associated with the haustorial development in T. chinense, DAPs were subjected to the KEGG pathway database. During the early developmental stage (FB), proteins with increased abundance were mainly involved in the pathways related to metabolic pathways (108 DAPs), photosynthesis (20 DAPs), and carbon fixation in photosynthetic organisms (12 DAPs). Proteins with decreased abundance were involved in only three pathways, namely ribosome (52 DAPs), systemic lupus erythematosus (3 DAPs), and flavonoid biosynthesis (3 DAPs). During the later developmental stage (FD), proteins with increased abundance were mainly involved in pathways related to metabolic pathways (158 DAPs), photosynthesis (23 DAPs), glyoxylate metabolism, dicarboxylate metabolism (15 DAPs), and phenylpropanoid biosynthesis (14 DAPs). In addition, the proteins whose abundance decreased were also associated with only three following pathways, ribosome (67 DAPs), systemic lupus erythematosus (4 DAPs), and alcoholism (4 DAPs) (Figure 3).

### 3.4. DAPs Involved in the Phytohormone Signaling Pathway

Based on the proteomic analysis, we observed that the expression levels of 17 proteins associated with the phytohormone signaling pathway were altered, including auxin-binding protein, mitogen-activated protein kinase, ABC transporter protein, and gibberellin-regulated protein (Table 1). We found that the majority of DAPs related to auxin signaling pathways were significantly increased during haustorial development in T. chinense, with the highest ratio of 2.34 for phosphoinositide phospholipase C (Table 1). Only one uncharacterized protein was significantly downregulated with a ratio of 0.47 and 0.37, respectively. Furthermore, all four proteins associated with the abscisic acid signaling pathway were significantly upregulated with a ratio that ranged from 1.57 to 2.58. In addition, three DAPs associated with the gibberellin signaling pathway abundance was marked downregulated, with the lowest ratio of 0.087 being observed for GAST-like protein after 10 days of haustoria development.

### 3.5. Phytohormone Concentrations

**Figure 4:**
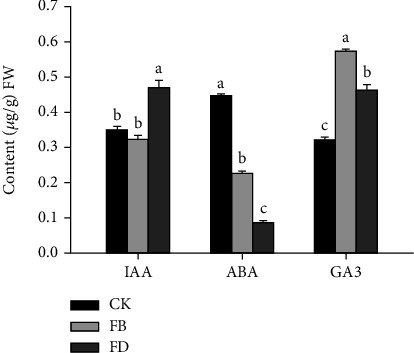
Average phytohormone concentrations of IAA, ABA, and GA3 in FB, FD, and control plants. Different letters above the bars indicate significant differences, *P* < 0.05.

To elucidate the correspondence between the level of hormones and the abundance of proteins, the IAA, ABA, and GA contents were analyzed using LC-MS-based analysis. The accumulation of IAA was slightly decreased after 10 days of haustorial development. However, the content of IAA was significantly increased after 20 days of haustorial development compared to the CK and FB. In contrast, the levels of ABA gradually decreased with the haustorial development. Interestingly, GA3 contents were initially upregulated, but then downregulated, reaching their highest level at 10 days (Figure 4).

### 3.6. Transcriptional Expression Analysis by qRT-PCR

**Figure 5:**
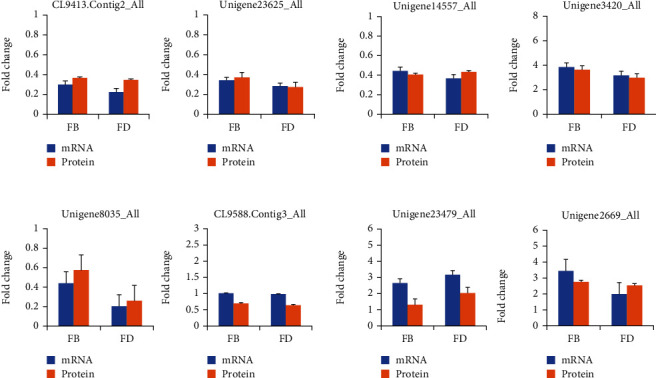
Analysis of the corresponding transcript levels of proteins by qRT-PCR.

To validate the correlation between the transcript level of mRNA and the abundance of proteins, a transcriptional analysis of 8 DAPs was analyzed by qRT-PCR. The results showed that the expressions of these genes were similar to those of the abundance of their corresponding protein. The results indicate the authenticity of the method used to confirm DAPs in our study (Figure 5).

## 4. Discussion

Branches and leaves of T. chinensis are widely used in traditional Chinese medicine for the treatment of rheumatism, hypertension, and obesity as well as for preventing miscarriages [24]. The haustorium is an essential parasitic organ developed by T. chinensis to penetrate host tissues. However, the molecular mechanism of T. chinensis haustorium development is still unclear. To gain insights into protein changes during the developmental reprogramming of haustoria formation in T. chinensis, comparative proteomic analysis based on the iTRAQ was performed. A total of 563 and 785 DAPs were successfully identified in the early and later developmental stages, respectively. These DAPs were functionally classified according to their roles and found to be enriched in the metabolic pathways, ribosome, phenylpropanoid biosynthesis, and phytohormone signaling pathway. Current results lay a foundation for the further identification and functional characterization of haustorial proteins in T. chinensis.

Previously, few studies have uncovered metabolic pathways that have an important role in haustorial development. Ichihashi et al. (2018) proposed that the accumulation of very long chain fatty acid (VLCFAs) involved in the developmental reprogramming of Thesium chinense haustoria formation in the natural environment [25]. Besides, some metabolites have been demonstrated response to haustorial development. For example, lignin-related compounds induced haustoria formation in P. japonicum and S. hermonthica with different specificities. High concentrations of lignin polymers induced haustorium formation. Treatment with laccase, a lignin degradation enzyme, promoted haustorium formation at low concentrations [26]. In the current study, 15 upregulated DAPs involved in phenylpropanoid biosynthesis were identified. Phenylpropanoid metabolism is one of the most important metabolic pathways, contributing to plant development [27]. The lignin biosynthetic pathways are known to be originated from the general phenylpropanoid pathway [28]. Together, these results imply that the accumulation of lignin leading to the T. chinensis haustorium formation was regulated by key proteins belonging to the phenylpropanoid metabolic pathway.

Phytohormones, such as IAA, GAs, and ABA, play crucial roles in regulating plant growth, development, and response to various stresses as previously reported [29, 30]. Auxin was demonstrated to be involved in the formation of cluster roots (CR) and adventitious roots (AR) [31]. Genes related to the auxin singling pathway were significantly enriched in the parasitic plant T. chinense and C. australis. [32]. In the meantime, genes involved in the polar auxin transport were enriched during haustorial and prehaustorial stages compared to the reference tissues, stem, and seedlings of dodder [33]. Polar auxin transport promotes the formation of local auxin maxima and gradients within tissues, resulting in the formation of patterns during cell division and differentiation in the root meristem [34]. The accumulation of auxin levels was also observed during haustoria initiation in sandalwood [15]. The current results demonstrated that the content of auxin was significantly increased after 20 days of haustorial development, which was consistent with the expression of proteins related to auxin abundance. GA3 was also considered an important regulatory factor in the haustorial development of parasitic plants, which may be originated from the effects of GA3 on xylem formation and elongation [35]. Endogenous GA3 was involved in the tracheary element differentiation in the Zinnia elegans xylogenic culture [36]. In parasite-host associations, ABA is considered a root-derived signaling molecule. In general, ABA levels in the parasitic roots were higher than in the host root, possibly to keep the stomata closed and the hydraulic conductivity of in roots response to higher transpiration [12]. However, the current study suggested that the levels of ABA gradually decreased during haustorial development. A more precise mechanism of the ABA function needs further investigation. In the meantime, we observed that the proteins associated with ABA signaling were significantly upregulated. We speculate that these proteins may act as negative regulators to control ABA biosynthesis. Altogether, these results imply that endogenous auxin, GAs, and ABA may function as important regulatory factors during the haustorial development in T. chinense.

## 5. Conclusions

n conclusion, we studied the protein and phytohormones profiles during the Taxillus chinensis haustoria development. Some crucial proteins involved in haustorial development were successfully identified in this study. More importantly, the findings of this study will improve our understanding of parasitism and contribute to the breeding program of Taxillus chinensis. We believe that further functional analysis focusing on the single crucial proteins involved in the developmental process is a critical need for the future. That will provide more precise information about the mechanisms underlying haustorial development in Taxillus chinensis.

## Figures and Tables

**Table 1:** The details information of differential abundant proteins (DAPs) related to phytohormone.

Protein name	Description	Subcellur location	FB-vs.-CK fold change	FD-vs.-CK fold change	Regulated stage	Pathway
A9PJ84	FHA domain-containing protein	Chloroplast	1.482	1.951	Up	ABA
M5W5K7	Uncharacterized protein	Nuclear	1.575	2.577	Up	ABA
D7TKF5	ABC-type xenobiotic transporter	Plasma membrane	1.656	1.967	Up	ABA
F6HUI4	Amine oxidase	Chloroplast	1.437	1.639	Up	ABA
Q52QX4	Auxin-repressed protein-like protein ARP1	Nuclear	1.753	1.655	Up	IAA
A7Y2G2	Germin-like protein	Cytosol	0.845	1.571	Up	IAA
B9RZ84	Glutaredoxin	Chloroplast	1.610	1.826	Up	IAA
A5BL97	Uncharacterized protein	Cytosol	1.753	1.882	Up	IAA
I3SDZ1	Uncharacterized protein	Cytosol	0.473	0.366	Down	IAA
F6I592	Ge1_WD40 domain-containing protein	Chloroplast	1.590	1.956	Up	IAA
Q6TKQ8	Mitogen-activated protein kinase	Chloroplast	1.678	1.674	Up	IAA
A5C0R5	Phosphoinositide phospholipase C	Chloroplast	1.881	2.340	Up	IAA
D7TWG7	RING-type domain-containing protein	Chloroplast	1.194	1.892	Up	IAA
Q0PN10	Glutathione S-transferase	Cytosol	1.661	1.456	Up	IAA
EEE76641	Predicted protein	Nuclear	1.568	1.355	Up	IAA
B3V944	GAST-like protein	Extracellular	0.087	0.119	Down	GA3
D3G6C0	Snakin-1	Extracellular	0.498	0.581	Down	GA3
M5XGG8	Gibberellin regulated protein	Chloroplast	0.190	0.137	Down	GA3

## Data Availability

The mass spectrometry proteomics data have been deposited to the ProteomeXchange Consortium via the PRIDE partner repository with the dataset identifier PXD029830.

## Supplementary Materials

Supplementary 1 Figure S1: the number of spectrums, peptides, and proteins identified from iTRAQ proteomics.

Supplementary 2 Table S1: primers sequences used for qRT-PCR validation.
