# Supplementary figures and images for: Comparative Proteomic Analysis Provides New Insights into the Development of Haustorium in Taxillus chinensis (DC.) Danser
Source: Biomed Res Int. 2022 Jul 28;2022:9567647. doi: 10.1155/2022/9567647 (PMC9356245; doi:10.1155/2022/9567647)

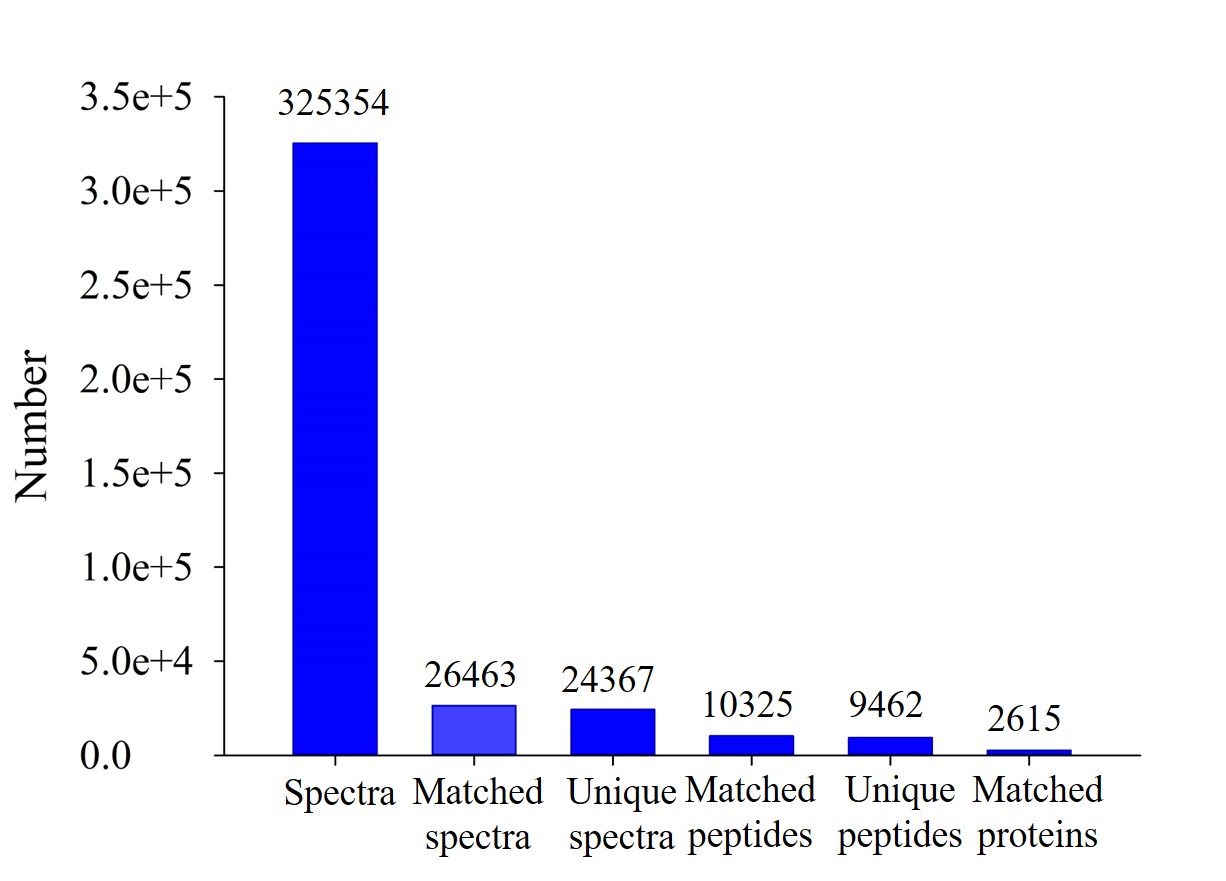

Supplement: Supplementary 1 — Figure S1: the number of spectrums, peptides, and proteins identified from iTRAQ proteomics. [file 9567647.f1.jpg]
