# Supplementary material for: Comparative Proteomic Analysis Provides New Insights into the Development of Haustorium in Taxillus chinensis (DC.) Danser
Source: Biomed Res Int. 2022 Jul 28;2022:9567647. doi: 10.1155/2022/9567647 (PMC9356245; doi:10.1155/2022/9567647)
Supplement: Supplementary 2 — Table S1: primers sequences used for qRT-PCR validation. [file 9567647.f2.pdf]

Table S1 Primers sequences used for qRT-PCR validation

actin-3-F	GGTCGTGACCTCACAGATGCTC
actin-3-R	GCTCCTGCTCATAGTCAAGAGC
CL9413.Contig2_All-F	TGCCGGATGGACTCTTCT
CL9413.Contig2_All-R	AATGCCACCAGCCACAAT
Unigene23625_All-F	TAAGCCTATTCCTGACCT
Unigene23625_All-R	CTTTGAATCCTCCTTGTG
Unigene14557_All-F	TCTTCTTCACCCATTACG
Unigene14557_All-R	CAACGCCTTTGATTCCTA
Unigene3420_All-F	TCTTCTTAGCGGTGGTGT
Unigene3420_All-R	TCTCAACTGACCCATCCT
Unigene8035_All-F	TTTACCCTCAAAGACCTG
Unigene8035_All-R	TGCAAGAGTGAGCCTAAT
CL9588.Contig3_All-F	CCTCTGAGCTAAGTGGGAAAG
CL9588.Contig3_All-R	CCGAAGTTACGGAAATGG
Unigene23479_All-F	ATCATCACCAGGCTGCGT
Unigene23479_All-R	TGCCTTGATGCCTCCACA
Unigene2669_All-F	TGTTATTGTAAGCCTGCTA
Unigene2669_All-R	TTGGGAACCTCATCATAG
